# FDG-PET/CT activity leads to the diagnosis of unsuspected TB: a retrospective study

**DOI:** 10.1186/s13104-018-3564-6

**Published:** 2018-07-13

**Authors:** Carolina Geadas, Carlos Acuna-Villaorduna, Gustavo Mercier, Mary B. Kleinman, C. Robert Horsburgh, Jerrold J. Ellner, Karen R. Jacobson

**Affiliations:** 1 Section of Infectious Diseases, Department of Medicine, Boston Medical Center and Boston University School of Medicine, 801 Massachusetts Ave, 2nd Floor, Boston, MA 02118 USA; 2 Section of Molecular Imaging and Nuclear Medicine, Department of Radiology, Boston Medical Center and Boston University School of Medicine, Boston, MA USA; 3 Infectious Disease Prevention and Health Services Bureau, Prevention and Health Promotion Administration, Maryland Department of Health and Mental Hygiene, Baltimore, MD USA; 4 Department of Epidemiology, Boston University School of Public Health, Boston, MA USA

**Keywords:** Latent, Subclinical TB, Imaging, Biomarkers, Risk

## Abstract

**Objective:**

*Mycobacterium tuberculosis* infection leads to latent or active tuberculosis (TB). Increased uptake on ^18^F-fluoro-2-deoxy-glucose-positron emission tomography/computed tomography (FDG-PET/CT) has been reported in the lungs and lymph nodes of individuals with recent infection and active TB, but not in individuals without known recent exposure or suggestive symptoms. We describe five patients with lung nodules not suspected to be due to TB in whom abnormalities on FDG-PET/CT scans ultimately were attributed to TB infection.

**Results:**

Patient records were searched using the words “positron emission tomography/computed tomography” and 24 codes for TB between 2004 and 2013. Patients with a diagnosis of TB and a PET/CT scan were included. Clinical and radiographic data were retrieved. PET/CT images were reviewed by an experienced radiologist. FDG-PET/CT scans revealed elevated FDG-uptake in lungs of five patients subsequently diagnosed with active (n = 3) or clinically inactive (n = 2) tuberculosis. Uptake magnitude was unrelated to disease activity. These findings suggest that tuberculosis latency may include periods of percolating inflammation of uncertain relationship to future disease risk.

## Introduction

The idea that *Mycobacterium tuberculosis* (MTB) infection exists in two discrete states—metabolically-inactive latent tuberculosis infection (LTBI) and metabolically-active tuberculosis (TB) disease—has recently been challenged with a new model of fluctuating activity ranging from quiescent to metabolically-active lesions [1, 2]. The American Thoracic Society (ATS) classifies persons exposed to and/or infected with MTB into distinct classes: [3] Class 1 comprises individuals with a history of exposure to TB but no evidence of infection; Class 2 includes individuals with evidence of LTBI but not of active disease; persons with Class 3 have clinically active disease; and Class 4 includes those with either a history of previous clinically active TB or a history of exposure to TB with abnormal stable findings on chest radiography, without clinically active disease.

^18^F-fluoro-2-deoxy-glucose-positron emission tomography/computed tomography (FDG-PET/CT) is a noninvasive imaging tool used primarily for cancer diagnosis and staging. It identifies areas of active inflammation by mapping where cells with high metabolic demand take up the radioactively-labeled glucose analogue (FDG). The radiotracer accumulates within inflammatory cells such as macrophages and neutrophils and can be quantified as a standardized uptake value (SUV). Previous reports have shown increased FDG-uptake in the lungs and lymph nodes of persons presumed to be recently infected with TB (household contacts of TB cases with a positive QuantiFERON-TB Gold) [4], in asymptomatic HIV co-infected individuals in a high TB burden setting [5], and in individuals with active TB lesions [6, 7]. FDG-uptake has variably risen or fallen in response to treatment [7]. Previous studies have investigated activity in persons with recent or ongoing TB exposure or in the process of TB disease diagnosis or treatment [8]. We identified five patients with TB infection [per positive tuberculin skin test (TST) or interferon-gamma release assay (IGRA)], but not suspected to have TB disease, that underwent FDG-PET/CT scan for evaluation of pulmonary nodules and had the final pathologic and/or microbiologic diagnoses of TB.

## Main text

### Methods

This retrospective study was conducted at Boston Medical Center (BMC)—a safety-net hospital with a large immigrant patient population. We searched the BMC clinical database using the words “positron emission tomography/computed tomography” in combination with 24 ICD-9 codes for TB. Patients that had both a FDG-PET/CT scan and a TB diagnosis were included if: (1) results were between January 2004 and December 2013 and (2) a diagnosis of Class 3 or 4 TB (per ATS standard classification [3]) was made within 90 days of a FDG-PET/CT. Medical records of included patients were abstracted. FDG-PET/CT images were reviewed by a nuclear radiologist (GM) who was blinded to the diagnoses. The study was approved by the BMC Institutional Review Board (IRB).

### Results

We identified 159 patients whose medical records included both a FDG-PET/CT scan and TB ICD-9 code. One hundred fifty-two were excluded: eight had a TB disease diagnosis prior to the scan, eight had only a history of TB exposure without evidence of infection (Class 1 TB), and 136 had a history of LTBI with no evidence of clinically active TB disease (Class 2 TB)—these individuals received alternative diagnoses for their pulmonary nodules.

We identified seven patients whose FDG-PET/CT scans showed pulmonary abnormalities that were ultimately attributed to TB. This included three cases of culture-proven TB (Class 3) and four cases of inactive TB (Class 4) who had evidence of LTBI and abnormal findings on PET/CT, but no clinical, microbiological or radiographic evidence of active disease. For two of the patients identified as having Class 4, wedge resections were not performed and therefore a diagnosis could not be unequivocally confirmed by histopathology. For this reason, these two patients were excluded from this evaluation.

**Fig. 1:**
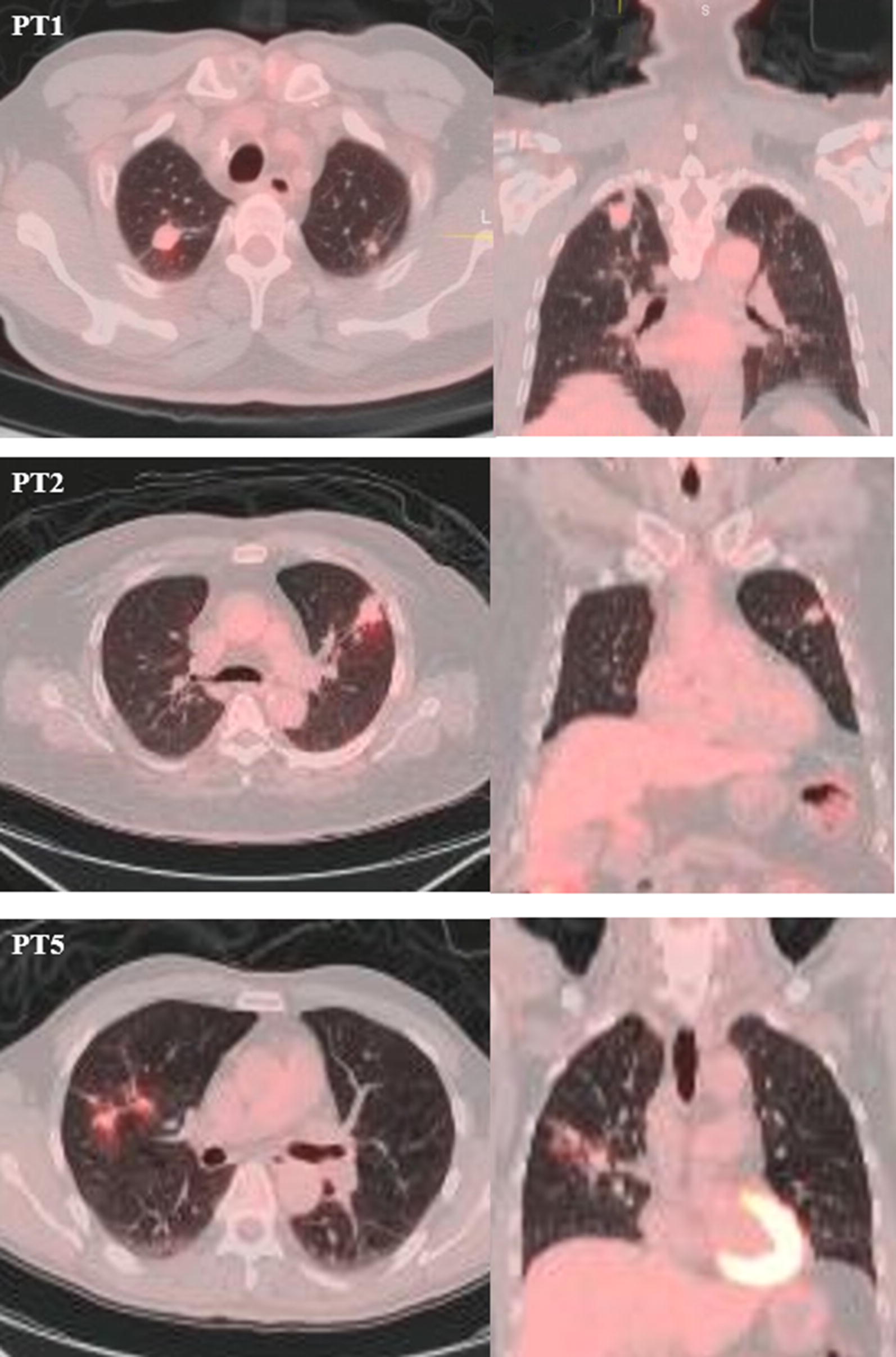
FDG-PET/CT scans of patients (PT) 1, 2, and 5. Images are axial and coronal fused PET/CT images with CT in standard lung windows and the PET in an orange color scale. PT1 shows a 2 cm spiculated lung nodule in the right upper lobe (RUL) with SUV_max_ of 2.72. PT2 shows a similar finding in the left upper lobe (LUL) but tracer uptake is slightly higher with SUV_max_ of 3.9. PT5 shows two RUL nodules, with 1.6 and 1.4 cm, both with SUV_max_ of 2.6

The three patients with culture-positive active (Class 3) TB (Table 1) were males from high TB burden countries (immigrated to the US in 1988, 1998, and 2010). Mean age was 55 years (range 34–77). One patient was HIV-infected (CD4 = 26 cubic cells per millimeter, not on antiretrovirals). Two had symptoms consistent with TB (cough, weight loss, night sweats) and one had had a self-resolved respiratory infection. All three patients showed abnormal uptake by upper lobe nodules on lung PET/CT scan. SUV_max_ ranged from 1.5 to 3.1 (Fig. 1). In addition to pulmonary TB, one patient had extra-pulmonary disease confirmed by a positive MTB culture of a colonic ulceration. In all three patients, TB was microbiologically confirmed by positive microscopy, culture, and/or polymerase chain reaction (PCR) of the sputum or bronchoalveolar lavage (Table 1).

**Table 1:** Characteristics of five patients who received a diagnosis of TB after FDG-PET/CT scan

Patient information	TB infection diagnosis	Comorbidities	Scan date	Thoracic CT scan	Thoracic FDG-PET scan	Microbiology and histopathology	Diagnosis and management
1. 69-year-old male from Morocco (immigrated to the US in 2004). Asymptomatic. Evaluated for potential lung metastases of prostate cancer	TST = 20 mm	DM, recently diagnosed prostate cancer (not on treatment)	2011	2 cm spiculated nodule in RUL, 5 mm nodule in LUL	RUL nodule uptake (SUV_max_ 2.72)	Negative sputum microscopy and culture. Wedge resection showed necrotizing granulomas	Class 4 TB. RIF and INH for 4 months
2. 58-year-old male from the Philippines (immigrated to the US in 2010). Asymptomatic. Evaluated for TB per immigration requirements	TST = 20 mm	DM	2011	2.1 cm spiculated nodule in LUL	LUL nodule uptake (SUV_max_ 3.9)	Negative sputum microscopy and culture. Wedge resection showed necrotizing granulomas	Class 4 TB. RIF and INH for 4 months
3. 34-year-old male from Haiti (immigrated to the US in 1998). Cough and night sweats for 1 month	Negative TST (self-reported)	HIV infection (CD4 cell count of 26 cells/mm, not on ART), smoking	2009	Centrilobular tree-in-bud opacities, 1 and 1.5 cm cavitary nodules in the LUL	LUL nodule uptake (SUV_max_ 3.1)	Positive MTB culture from sputum (at 3 weeks) and from bronchoalveolar lavage (at 3 days)	Class 3 TB. RIPE
4. 77-year-old male from the Dominican Republic (immigrated to the US in 2010). Unintentional weight loss. Known to have an abnormal chest X-ray	TST = 15 mm (negative 8 weeks prior)	COPD	2013	Scarring of LUL, multiple nodules and tree-in-bud lesions in RUL, right hilar calcifications	RUL nodule uptake (SUV_max_ 1.5). Left hilar uptake (SUV_max_ 2.3). Colon uptake (SUV_max_ 7.9)	Positive MTB PCR from sputum. Positive MTB culture from colon ulceration (found on colonoscopy)	Class 3 TB. RIPE
5. 54-year-old male from Vietnam (immigrated to the US in 1988). Cough for 2 weeks, which resolved spontaneously	Positive TST (self-reported) TB disease treated in 1979; LTBI treatment in 1989 upon arrival to the US	COPD	2011	1.6 and 1.4 cm nodules in the RUL	RUL nodules uptake (SUV_max_ 2.6)	Positive MTB culture from bronchoalveolar lavage (at 5 weeks)	Class 3 TB. RIPE

*CT* computerized tomography, *FDG-PET* ^18^F-fluoro-2-deoxy-glucose-positron emission tomography, *TST* tuberculin skin test, *DM* diabetes mellitus, *RUL* right upper lobe, *LUL* left upper lobe, *SUV*_*max*_ maximum standard uptake value, *TB* tuberculosis, *RIF* rifampin, *INH* isoniazid, *HIV* human immunodeficiency virus, *MTB Mycobacterium tuberculosis*, *RIPE* rifampin, isoniazid, pyrazinamide and ethambutol, *COPD* chronic obstructive pulmonary disease, *PCR* polymerase chain reaction

The two individuals with culture-negative, inactive (Class 4) TB (Table 1) were both males from high TB burden countries as well (immigrated to the US in 2004 and 2010). Mean age was 63.5 years (range 58–69). Both patients had diabetes. Both were asymptomatic. On PET/CT scan, both showed increased FDG-uptake by upper lobe nodules (Fig. 1). SUV_max_ ranged from 2.72 to 3.9. Neither had clinical evidence of extra-pulmonary disease by symptoms or on PET/CT. Both had negative sputum microscopies and cultures. Wedge resection of the areas with increased uptake was performed and in both cases necrotizing granulomas were identified on histopathology; this tissue was also negative for acid fast bacilli (AFB) smears and mycobacterial cultures did not grow (Table 1).

All five patients were subsequently treated with isoniazid and rifampin or with a four-drug regimen of isoniazid, rifampin, pyrazinamide, and ethambutol per standard ATS guidelines.

### Discussion

Although increased PET activity has been described in cases of active TB and recent TB infection, the finding of increased activity in asymptomatic individuals without recent exposure has not been reported. We found two asymptomatic LTBI patients who had wedge resections in the region of increased FDG-uptake that revealed granulomas, indicating that TB was driving the metabolic activity. The presence of metabolic activity in individuals without symptoms or known recent TB exposure supports the concept that TB latency is not static; metabolic activity may fluctuate during latency driven by a low-level replicating mycobacterial burden as demonstrated by our patients’ negative cultures and lack of constitutional symptoms. It is unclear whether in the absence of treatment these foci would regress spontaneously or progress to active disease. Autopsy studies, in fact, have revealed evidence of minimally active TB in patients that died from other causes and still were asymptomatic [2]. A lesson of HIV infection is that viable organisms exist within latent foci and have the potential to reactivate when no longer suppressed [9]. Cell wall synthesis of bacilli also must occur during latency as isoniazid, a bactericidal cell-wall active drug that acts on replicating bacilli, successfully prevents progression from LTBI to TB disease [2].

With its high sensitivity, PET/CT captures activity reflecting very low burden TB. A recent study reported that among HIV co-infected individuals with LTBI, those with lung abnormalities on FDG-PET/CT (including infiltrates, fibrotic scars, or active nodules) were more likely to subsequently develop symptomatic active disease than those without [5]. Interestingly the two asymptomatic patients identified in our study (patients 1 and 2) had SUV_max_ in the same range (2.7–3.9) as the three symptomatic patients (patients 3, 4, and 5) who had culturable TB (range 1.5–3.1), suggesting that these groups may be very close in the disease spectrum. It is notable, although difficult to explain, that despite similar inflammation the Class 3 cases were symptomatic whereas the Class 4 cases were not. A current study investigating the natural history of FDG-avid pulmonary nodules in HIV-uninfected contacts of multidrug-resistant TB cases in Cape Town, South Africa (DMID protocol number 16-0112), should shed light on the rate of progression to active disease.

### Conclusions

Combined with our findings, the current studies indicate that symptom screening and sputum smear and mycobacterial culture are limited in their ability to detect TB activity, including latency. Improved biomarkers are needed to stratify individuals according to risk of progression to active disease. FDG-PET/CT may reflect an early event in TB reactivation and could be of value for biomarker development. Our observations raise two important points in clinical practice. The first is that TB may produce very similar findings to lung cancer on FDG-PET/CT, making it essential to keep TB in the differential diagnosis even in patients without a known recent exposure or classic symptoms. The second is that, in patients with remote exposure to TB or a known history of LTBI (per positive TST or IGRA), FDG-PET/CT activity may reflect low-burden TB disease with imminent reactivation, prompting treatment.

## Limitations

A limitation of our study is that none of our patients had follow-up PET/CT scans to determine whether FDG activity resolved after treatment. In a series of recently infected persons, increased lymph node activity diminished in three of four patients after treatment of LTBI [4]. Additionally, quantitative changes in FDG-uptake two months after starting treatment were associated with long term outcomes in a cohort of multidrug-resistant TB patients receiving treatment in South Korea [10]. On the other hand, most HIV-uninfected patients with drug-sensitive TB treated in South Africa had persistent PET/CT activity at the time of sputum culture conversion and even up to one year after [11]. It is therefore unclear whether the foci of FDG activity described in our study would have regressed spontaneously or progressed to active disease without treatment.

## Abbreviations

ATS: American Thoracic Society
CT: computerized tomography
FDG-PET: ^18^F-fluoro-2-deoxy-glucose-positron emission tomography
HIV: human immunodeficiency virus
IGRA: interferon-gamma release assay
LTBI: latent tuberculosis infection
PCR: polymerase chain reaction
SUV: standard uptake value
TB: tuberculosis
TST: tuberculin skin test
